# Association Study of *MTHFR* C677T Polymorphism and Birth Body Mass With Risk of Autism in Chinese Han Population

**DOI:** 10.3389/fpsyt.2021.560948

**Published:** 2021-02-25

**Authors:** Jishui Zhang, Xueqian Ma, Yi Su, Lifang Wang, Shaomei Shang, Weihua Yue

**Affiliations:** ^1^Department of Mental Health, Beijing Children's Hospital, Capital Medical University, Beijing, China; ^2^National Center for Children's Health, Beijing, China; ^3^School of Nursing & Sixth Hospital, Peking University, Beijing, China; ^4^Peking University Sixth Hospital, Institute of Mental Health, Beijing, China; ^5^National Clinical Research Center for Mental Disorders (Peking University Sixth Hospital), Beijing, China; ^6^National Health Commission (NHC) Key Laboratory of Mental Health, Research Unit of Diagnosis and Treatment of Mood Cognitive Disorder (2018RU006), Chinese Academy of Medical Sciences, Beijing, China; ^7^School of Nursing, Peking University, Beijing, China; ^8^PKU-IDG/McGovern Institute for Brain Research, Peking University, Beijing, China

**Keywords:** autism, MTHFR gene, polymorphism, birth body mass, risk of disease

## Abstract

**Objective:** To explore the association of the methylenetetrahydrofolate reductase (*MTHFR*) C677T polymorphism with birth body mass and risk of autism in Chinese Han population.

**Methods:** A total 1,505 Chinese Han autism patients were recruited, using the Diagnostic and Statistical Manual of Mental Disorders, 4th revised version (DSM-IV-R) diagnostic criteria for autism, and 1,308 sex-matched healthy controls were also enrolled for the study. All the participants' birth body masses were counted according to the medical records. The *MTHFR* C677T genotypes were detected using the polymerase chain reaction-restrict fragment length polymorphism (PCR-RFLP) method. The association between C677T polymorphism, birth body mass, and risk of autism were analyzed using the chi-square tests.

**Results:** The present study found that the *MTHFR* 677T was significantly associated with risk of autism [*P* = 0.004, odds ratio (*OR*) = 1.18, 95% *CI* = 1.02–1.29). The autism children more frequently showed low birth body mass (<2.5 kg) than healthy control subjects (8.6 vs. 5.3%, *P* = 0.001, *OR* = 1.67, 95% *CI* = 1.24–2.26). The interactive effects between *MTHFR* 677T and low birth body mass (*P* = 0.0001, *OR* = 2.18, 95% *CI* = 1.44–3.32) were also significantly associated with risk of autism.

**Conclusions:** The *MTHFR* C677T polymorphism and low birth body mass may be associated with risk of autism in Chinese Han population.

## Introduction

Autism is a common neurodevelopmental disorder, which is clinically characterized by social communication and language communication disorders and repetitive and stereotyped interests and hobbies. At present, exact molecular and biochemical mechanism of autism has not been fully understood. Nevertheless, multiple genetic factors, environmental factors, and their interactions are believed to be involved in autism (1–4).

A large number of family and twin studies show that autism is highly inheritable, with heritability ranging from 60 to 90% (5–8). Genetic risk factors are considered to explain most risks of autism. In a large-scale cross-country cohort study of more than two million people carried out by Ban et al. (9), 22,156 of them were diagnosed with autism, with heritability of ~80% but without support for maternal effect and environmental impact, indicating that the changes in the occurrence rate of autism in this population were mainly attributed to genetic impact. However, new evidence show that environmental factors also increase the risk of autism, such as prenatal estrogen, maternal body mass index (BMI), perinatal complications, birth season, birth body mass, and other environmental risk factors, as well as the interaction between heredity and environment, which are also related to the risk of autism (10–16).

A literature reports that the risk of autism may be related to a variety of key genes involved in neurodevelopmental regulation, among which methylenetetrahydrofolate reductase (*MTHFR*) gene may be one of the candidate genes for neurodevelopmental disorders such as autism (17). The *MTHFR* is a key enzyme of folate metabolism in the process of one-carbon metabolism, converting 5,10-methylenetetrahydrofolate into 5-methyltetrahydrofolate, and participating in folate and homocysteine conversion correlated to DNA methylation (18). The active form of *MTHFR* could impact on the generation of 5-methyltetrahydrofolate, which is the active form of folate *in vivo*. Folate metabolism plays an extremely important role in the risk of neurodevelopmental disorders such as autism (19, 20). In a case–control cohort study of 45,300 offspring by Levine et al. (21), folate supplementation before and/or during pregnancy was observed to reduce the risk of autism in offspring. Methylation is a common regulation process of gene expression that influences cellular development and function (22), which is dependent on S-adenosylmethionine as a methyl donor. S-Adenosylmethionine originated from methionine cycle in which 5-methyltetrahydrofolate transfers methyl groups to homocysteine in a reaction catalyzed by methionine synthase to produce methionine (23).

The *MTHFR* gene has been identified as having 14 common or rare single nucleotide polymorphisms related to enzyme deficiency (24), of which C677T (rs1801133) located in exon 5 of the gene is one of the most reported (25), and its polymorphism TT genotype leads to a decrease in enzyme activity, which is only 40–50% of the CC-type enzyme activity at 37°C (17, 26). As consequences of polymorphism of MTHFR, reduction in MTHFR enzymatic activity would cause impaired methylation, deficiency of folate, as well as increase in homocysteine. Individuals with the 677T *MTHFR* alleles are predisposed to homocysteinemia and low plasma folate (27) and DNA hypomethylation (28). Individuals with the 677TT genotype manifest the highest homocysteine and lowest plasma folate levels (29) and lowest DNA methylation (27, 30). Children with autism have been found to have high plasma levels of homocysteine (31) and a biochemical profile of reduced methylation capacity (32). High levels of plasma homocysteine and increased oxidative stress have generally been associated in the pathophysiology of autism (33).

However, whether the above environmental factors and genetic susceptibility increase the risk of autism synergistically still needs further verification. This study intends to explore the association between *MTHFR* gene C677T polymorphism, birth body mass, and risk of autism in Chinese Han population.

## Materials and Methods

### Participants

This study included a total of 1,505 autistic children of Han nationality in China, including 998 boys and 507 girls. Admission criteria were the following: meet the diagnostic criteria for autism in the 4th edition of the Diagnostic and Statistical Manual of Mental Disorders (DSM-IV-TR), Han nationality, regardless of gender, age 3–18 years old, outpatient children, and with written informed consent form signed by the children themselves or their guardians. Exclusion criteria were the following: patients with mental retardation, schizophrenia, affective disorders, and other mental disorders that meet the DSM-IV Axis I diagnostic criteria; patients with severe physical diseases; and those unwilling to sign the informed consent form.

The control group consisted of 1,318 Chinese Han healthy controls, including 792 male and 526 female. Admission criteria were the following: Han nationality, regardless of gender, age 3–18 years old, database of normal subjects recruited from the community or physical examination center, with written informed consent form signed by the subjects themselves or their guardians. Exclusion criteria were the following: other mental disorders conforming to axis I diagnostic criteria such as autism, mental retardation, schizophrenia, and affective disorders in DSM-IV; patients with severe physical diseases; and those unwilling to sign the informed consent form.

### Methods

#### Birth Body Mass Statistics

All the participants' birth body masses were counted according to the medical records. According to the World Health Organization (WHO) standards, the birth body mass of full-term normal delivery children is divided into three groups: low birth body mass infants (<2.5 kg), normal birth body mass infants (2.5–4.0 kg), and fetal macrosomias (≥4.0 kg) (34).

#### Genomic DNA Extraction

Five milliliters of peripheral venous blood was extracted from all subjects, which was placed in ethylenediaminetetraacetic acid (EDTA) anticoagulation tube and stored at 4°C. A small amount of blood genomic DNA extraction kit from Qiagen Company was used to extract genomic DNA from samples within 1 week. After quality control, the samples were stored at −70°C for later use.

#### Genotype Detection of *MTHFR* C677T (rs1801133) Polymorphism Site

The PCR amplification primer sequences were as follows: upstream, 5′ AGC CCA GCC ACT CAC TGT TTT 3′; downstream, 5′ CAG CGA ACT CAG CAC TCC A 3′. Twenty-five microliters PCR amplification reaction system consist of 0.4 μM primer, 10 mM Tris–HCl (pH 8.3), 1.5 mM magnesium chloride, 50 mM potassium chloride, 200 μM deoxyribonucleotide triphosphates (dNTPs), 30–50 ng genomic DNA, and 1 U Taq DNA polymerase. PCR amplification conditions were predenaturation at 94°C for 5 min, denaturation at 94°C for 30 s, annealing at 64°C for 30 s, extension at 72°C for 1 min, a total of 35 cycles, and finally extension at 72°C for 10 min. The PCR products were digested with restriction enzyme *Hinf* I at 37°C overnight and observed by 4% agarose gel electrophoresis.

### Statistical Analyses

All data were managed and analyzed by Statistical Packages for Social Science 16.0 (SPSS 16.0) software. The Hardy–Weinberg equilibrium test was carried out on the genotype distribution of patients with fitness test. Chi-square analysis was used to test the association between the *MTHFR* C677T polymorphism site and birth body mass and risk of autism, and logistic regression analysis was used to comprehensively evaluate the contribution of genetic and environmental factors to autism risk. The statistical significance level of bilateral tests was set at *P* < 0.05.

## Results

### Hardy–Weinberg Equilibrium Test

The genotype frequency of *MTHFR* C677T (rs1801133) polymorphism in the case group and the control group both passed the Hardy–Weinberg equilibrium test, and there was no significant difference between the observed value and the expected value (*P* > 0.05) (Table 1). This showed that the research subjects were collected from a large population, and the individuals were randomly matched. There were no significant natural selection, migration, and other factors affecting the genetic balance, and the data were reliable.

**Table 1:** Hardy–Weinberg equilibrium test for *MTHFR* C677T (rs1801133) polymorphism.

		**Genotype**			**Chi square**	***P*-value**
		**CC**	**CT**	**TT**		
Case group	Actual number (O)	630	670	205	1.573	0.207
	Expected number (C)	618.75	692.49	193.75		
	(O – C)2/O	0.201	0.755	0.617		
Control group	Actual number (O)	618	550	150	2.613	0.102
	Expected number (C)	605.04	575.91	137.04		
	(O – C)2/O	0.272	1.221	1.120		

### Association Test of *MTHFR* C677T With Risk of Autism

The genotype and allele distributions are all shown in Table 2. There were statistically significant differences both in genotype frequencies (*x*^2^ = 8.088, df = 2, *P* = 0.018) and in allele frequencies (*x*^2^ = 8.251, df = 1, *P* = 0.004), respectively (Table 2). The results suggested that the risk allele 677T of *MTHFR* gene was significantly associated with risk of autism [odds ratio (*OR*) = 1.18, 95% *CI* = 1.02–1.29].

**Table 2:** Association analysis between *MTHFR* C677T polymorphism and risk of autism.

	**Case group** ** (*n* = 1,505)**	**Control group** ** (*n* = 1,318)**	**Chi square** ** (degree of freedom)**	***P*-value**
CC	630 (0.419)	618 (0.469)	8.088 (2)	0.018
CT	670 (0.445)	550 (0.417)		
TT	205 (0.136)	150 (0.113)		
C	1,930 (0.641)	1,786 (0.678)	8.251 (1)	0.004
T	1,080 (0.359)	850 (0.322)		

### The Correlation Analysis Between Birth Body Mass and Risk of Autism

**Figure 1:**
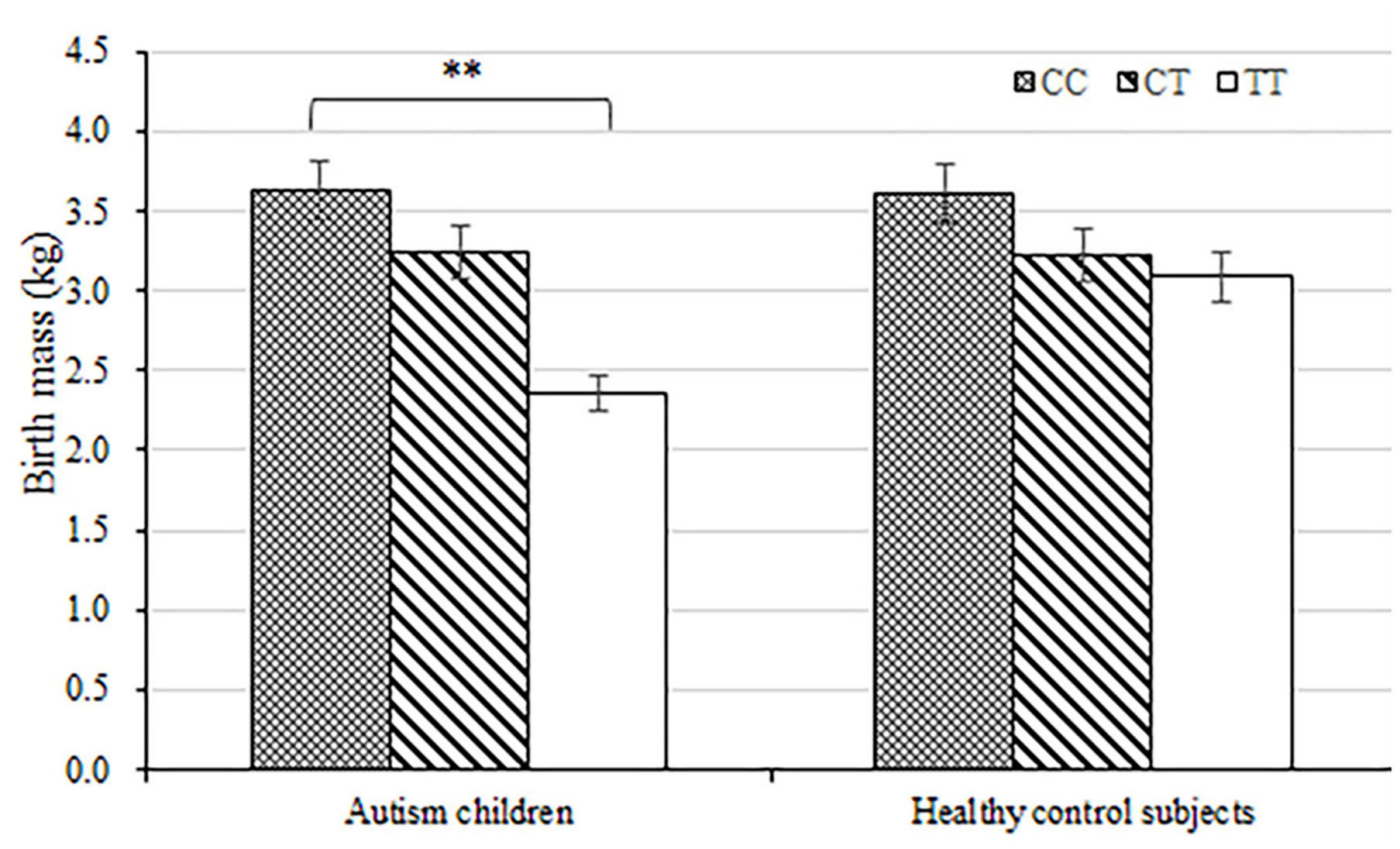
Load-dependent effects of *MTHFR* C677 genotypes on the birth body mass (kg) in autism children but not in healthy control subjects. Error bars ± 1.00 standard error. **P < 0.01.

The average birth body mass in autism group was (3.15 ± 0.74) kg, male (3.24 ± 0.33) kg, and female (3.06 ± 0.62) kg. The average birth body mass of the healthy control group was (3.24 ± 0.52) kg, male (3.35 ± 0.49) kg, female (3.22 ± 0.46) kg, respectively. According to the WHO standards, newborn body mass is divided into low birth weight infants <2.5 kg, normal birth weight infants 2.5–4.0 kg, and fetal macrosomia ≥4.0 kg, respectively. The frequency of autism children with low birth body mass in the autism group (8.6%) was higher than that in the healthy control group (5.3%) (*P* = 0.003) (Table 3 and Figure 1). Compared with non-low birth body mass infants, low birth body mass infants increased the effect value of autism risk (*OR* = 1.67, 95% *CI* = 1.24–2.26), suggesting that low birth body mass infants may increase risk of autism.

**Table 3:** Correlation analysis of birth body mass and risk of autism.

	**Case group** **(*n* = 1,505)**	**Control group** **(*n* = 1,318)**	**Chi square** **(degree of freedom)**	***P*-value**
Low birth body mass infants	129 (8.6%)	70 (5.3%)	11.399 (2)	0.003
Normal birth body mass infants	1,278 (84.9%)	1,159 (87.9%)		
Fetal macrosomias	98 (6.5%)	89 (6.8%)		

### The Interactive Effects of *MTHFR* 677T and Low Birth Body Mass on Risk of Autism

To explore the potential interaction effects of the risk allele 677T of *MTHFR* gene and low birth body mass on the risk of autism, the number of children with autism who both carried *MTHFR* 677T and low birth body mass, were counted then were compared to the other children. The results indicated that the frequency of autism children both carrying *MTHFR* 677T and with low birth body mass (*n* = 75, 5.0%) was statistically higher than that of other subjects (*n* = 30, 2.3%). Although the number of patients carrying risk factors of 677T allele and low birth body mass infants in the two groups was relatively small (*n* = 46 in total), there was also a strong statistical correlation effect (χ^2^ = 14.38, *P* = 0.0001; *OR* = 2.18, 95% *CI* = 1.44–3.32). The logistic multifactor regression analyses were conducted to explore the effects of potential risk factors for autism. The diagnosis of autism was set as dependent variable, and the independent variables includes age, gender, *MTHFR* C677T polymorphism, birth body mass, and interaction between *MTHFR* C677T polymorphism and birth body mass, respectively. The results showed that sex, *MTHFR* C677T polymorphism, birth body mass, and interaction between *MTHFR* C677T polymorphism and birth body mass all significantly increased the risk of autism (Table 4).

**Table 4:** Multivariate logistic regression analyses of risk factors of autism.

	**Coefficient**	***Chi square***	***P*-value**	***OR* (95% *CI*)**
Age	0.020	0.006	0.941	0.98 (0.57–1.57)
Gender	0.381	4.232	0.015	1.42 (1.09–2.13)
*MTHFR* C677T	−0.289	3.892	0.033	0.82 (0.48–0.97)
Birth body mass	0.295	3.921	0.029	1.24 (1.07–1.68)
*MTHFR* C677T × birth body mass	0.402	12.011	<0.001	2.57 (1.56–3.21)

## Discussion

The causes of autism, as a common neurodevelopmental disorder, are complex, containing both genetic and environmental factors. It has been reported in the literatures that multiple neurodevelopmental-related susceptibility genes might be associated with the risk of autism, such as neuregulin genes *NRX1/3, NLGN3/4*, etc. (35–38). The folic acid metabolic-rate-limiting enzyme, methylenetetrahydrofolate reductase (*MTHFR*), also may play an important regulatory role in neurodevelopmental disorders such as autism (19, 20). At the same time, there were also many reports that perinatal environmental factors such as low birth body mass may increase the risk of autism (10–16). The results of the present study also further confirmed some above-mentioned findings.

As one of the key enzymes in metabolic regulation, *MTHFR* is also one of the important candidate genes for common neurodevelopmental disorders. More than 10 mutations have been found contributing to effects of the *MTHFR* enzyme, of which C677T is one of the most common mutations found up to the present (24, 25). Decreased enzyme activity and heat tolerance caused by mutations can lead to folic acid metabolic abnormalities, methylation abnormalities, and neurodevelopmental disorders (17). This study suggested that carriers of *MTHFR* 677T allele were more likely to show an increased risk of autism than carriers of CC homozygotes (*P* = 0.004, *OR* = 1.18, 95% *CI* = 1.02–1.29), to some extent, which verifies that *MTHFR* may be one of the predisposing genes for autism. Paşca et al. (39) first reported the association between *MTHFR* C677T and risk of autism; however, it was a small sample study that just was carried out in 15 cases of autism and 25 cases of autism spectrum disorders. However, dos Santos (40) did not find any association between *MTHFR* C677T and risk of autism in Brazilian population. Sener et al. (41) also failed to find a significant association between *MTHFR* C677T and autism in a small sample population in Turkey (98 autism children and 70 healthy control subjects). Guo et al. (42) found that *MTHFR*C677T may be associated with risk of autism in a small Han Chinese sample of 186 autistic patients and 186 controls. It suggests that the association between *MTHFR* C677T and autism may be influenced by genetic heterogeneity among different populations. Shaik et al. (43) attempted to use six polymorphic loci of folate metabolism-related genes including MTHFR C677T in 138 pairs of autism spectrum disorders and control samples for artificial neural network calculation, and combined with meta-analysis of 1,361 autism spectrum disorders and 6,591 normal subjects, they finally found that C677T might be one of the important risk factors for autism.

In 2019, Sadeghiyeh et al. (44) in a meta-analysis of 18 studies evaluated the association between MTHFR 677C>T polymorphism and autism risk. Of the 18 studies, eight were conducted in Caucasian countries, three studies were in African population, and six studies were in Asian population. Their results suggested that MTHFR 677C>T was associated with increased autism risk in overall and by ethnicity among Caucasians, Asians, and Africans. In another recent meta-analysis by Razi et al. (45), 17 studies were included, and their results also suggested a significant association between C667T polymorphism and ASD risk in overall. However, in subgroup analysis, the significant association was only found among Caucasians but not in East Asians and Africans. The results of their subgroup analysis were inconsistent with the above meta-analysis (44), probably because only three studies were included in East-Asian countries and the sample size was insufficient. However, Sadeghiyeh et al. (44) also searched some Chinese databases, so there were more studies conducted in Asia. Therefore, the finding that there was a significant association between C677T and autism risk among Asians was more convincing, which is in line with our results as well.

Shaik Mohammad et al. (43) developed an artificial neural network (ANN) model as the predictors of autism risk using *GCPII* C1561T, *SHMT1* C1420T, *MTHFR* C677T, *MTR* A2756G, and *MTRR* A66G. The ANN model showed 63.8% accuracy in predicting the risk of autism. In addition to our research, many other studies on Chinese Han children also showed *MTHFR* C677T to be a genetic risk factor for autism (46–48). Thus, *MTHFR* C677T may be considered as a potential biomarker of children with autism. Future research can also use *MTHFR* C677T to develop a prediction model to evaluate its prediction accuracy.

In addition, this study also found that low birth body mass (<2.5 kg) might increase risk of autism. There have been many reports that low birth body mass may be one of the risk factors for autism (16, 49), and the meta-analysis by Hisle-Gorman et al. (16) also suggests that epilepsy, maternal mental history during pregnancy, low birth body mass, etc. may increase the risk of autism. Willfors et al. (50) found that the correlation between birth body mass and autism was not significant but showed a similar trend (β = −0.01, *P* = 0.05) in the analysis of 54 pairs of identical twins with inconsistent autistic characteristics, indicating that low birth body mass was related to the risk of autism. A meta-analysis reported by Wang et al. (51) investigated the risk factors of childhood autism before, during, and after birth, which also confirmed that the increased risk of autism was related to low birth body mass [relative risk (*RR*) = 1.26; 95% *CI*: 1.20–1.34; *P* < 0.001].

In our study, we further explore the interactive effects of MTHFR 677T and low birth body mass on the risk of autism. The results indicated that the frequency of autism children both carrying *MTHFR* 677T and with low birth body mass (*n* = 75, 5.0%) was much higher than that of other subjects (*n* = 30, 2.3%) (χ^2^ = 14.38, *P* = 0.0001; *OR* = 2.18, 95% *CI* = 1.44–3.32). Moreover, the logistic regression analysis showed that gender, *MTHFR* C677T polymorphism, birth body mass, interaction between *MTHFR* C677T polymorphism, and birth body mass significantly increased risk of autism in children. El-Baz et al. (52) found that low birth body mass increased the risk of autism in 31 cases of autism spectrum disorders and 39 healthy controls in Egypt, and the frequency of *MTHFR* 677T in case group was higher than that in healthy controls (31.1 vs. 5.13%) (*P* < 0.001), which was basically consistent with the conclusion of this study. A meta-analysis showed association between maternal *MTHFR* C677T polymorphism with low birth body mass under most of the genetic models, respectively. Specifically, the carriers of the TT genotype increased the risk of low birth body mass (53). One explanation is that when *MTHFR* C677T causes the decrease in enzyme activity, the plasma homocysteine concentration increases, which leads to oxidative stress, arteriolar constriction, endothelial damage, and placental thrombosis (54, 55). All these conditions might be associated with impaired flow and prothrombotic changes in the vessel wall, inadequate trophoblast invasion into the uterine vasculature, and placental hypoperfusion that subsequently triggers poor pregnancy outcomes including low birth body mass (56). Another explanation is that *MTHFR* enzyme activity is reduced due to *MTHFR* C677T, which may cause the deficiency of folate. Folate deficiency is a modifiable nutritional status that has been linked with adverse pregnancy outcomes including low birth body mass (57, 58). Thus, the gene–environmental interaction between MTHFR 677T risk allele and low birth body mass may further increase the susceptibility of autism. It further supports the pathogenesis reported in the literatures that autism may be a complex disease caused by the interaction of genetic and environmental risk factors (13–16).

In the present study, the mean age of healthy control subjects was much older than that of autistic patients. Although there was age mismatch, it could also ensure that there was no risk of autism in the control group, and the birth body mass statistics of normal subjects were relatively accurate. In the future, it is planned to further expand the sample size and include age-matched healthy controls. In addition, the specific neurobiological mechanism of genetic polymorphism affecting risk of autism needs to be further elaborated, and further verification of sample size expansion and long-term follow-up in different populations is needed. In conclusion, this study suggests that *MTHFR* 677T and low birth body mass may be one of the important influencing factors of autism risk.

## Data Availability Statement

The original contributions presented in the study are included in the article/supplementary materials; further inquiries can be directed to the corresponding author/s.

## Ethics Statement

The studies involving human participants were reviewed and approved by the medical ethics committee of Peking University Sixth Hospital and Beijing Children's Hospital. Written informed consent to participate in this study was provided by the participants' legal guardian/next of kin.

## Author Contributions

JZ, SS, and WY designed the study. JZ, SS, XM, and WY contributed to analysis, interpretation of data, and wrote the first draft of the manuscript. YS and LW contribute to the clinical assessment and experimental examination, as well as the statistical analyses. All authors contributed to drafting the work, revising it critically for important intellectual content, made substantial contributions to the concept, design of the study, acquisition, analysis, and interpretation of the data.

## Conflict of Interest

The authors declare that the research was conducted in the absence of any commercial or financial relationships that could be construed as a potential conflict of interest.
